# Covalently grafting first-generation PAMAM dendrimers onto MXenes with self-adsorbed AuNPs for use as a functional nanoplatform for highly sensitive electrochemical biosensing of cTnT

**DOI:** 10.1038/s41378-022-00352-8

**Published:** 2022-03-30

**Authors:** Xin Liu, Yong Qiu, Deming Jiang, Fengheng Li, Ying Gan, Yuxuan Zhu, Yuxiang Pan, Hao Wan, Ping Wang

**Affiliations:** 1 Biosensor National Special Laboratory, Key Laboratory for Biomedical Engineering of Education Ministry, Department of Biomedical Engineering, Zhejiang University, 310027 Hangzhou, China; 2 Cancer Centre, Zhejiang University, 310058 Hangzhou, Zhejiang China; 3 State Key Laboratory of Transducer Technology, Chinese Academy of Sciences, 200050 Shanghai, China; 4 Binjiang Institute of Zhejiang University, 310053 Hangzhou, China; 5 School of Biomedical Engineering, Tianjin Medical University, 300070 Tianjin, China; 6 Research Center of Smart Sensing, ZhejiangLab, 310027 Hangzhou, China

**Keywords:** Electronic properties and materials, Biosensors

## Abstract

2D MXene-Ti_3_C_2_T_χ_ has demonstrated promising application prospects in various fields; however, it fails to function properly in biosensor setups due to restacking and anodic oxidation problems. To expand beyond these existing limitations, an effective strategy to for modifying the MXene by covalently grafting first-generation poly(amidoamine) dendrimers onto an MXene in situ (MXene@PAMAM) was reported herein. When used as a conjugated template, the MXene not only preserved the high conductivity but also conferred a specific 2D architecture and large specific surface areas for anchoring PAMAM. The PAMAM, an efficient spacer and stabilizer, simultaneously suppressed the substantial restacking and oxidation of the MXene, which endowed this hybrid with improved electrochemical performance compared to that of the bare MXene in terms of favorable conductivity and stability under anodic potential. Moreover, the massive amino terminals of PAMAM offer abundant active sites for adsorbing Au nanoparticles (AuNPs). The resulting 3D hierarchical nanoarchitecture, AuNPs/MXene@PAMAM, had advanced structural merits that led to its superior electrochemical performance in biosensing. As a proof of concept, this MXene@PAMAM-based nanobiosensing platform was applied to develop an immunosensor for detecting human cardiac troponin T (cTnT). A fast, sensitive, and highly selective response toward the target in the presence of a [Fe(CN)_6_]^3−/4−^ redox marker was realized, ensuring a wide detection of 0.1–1000 ng/mL with an LOD of 0.069 ng/mL. The sensor’s signal only decreased by 4.38% after 3 weeks, demonstrating that it exhibited satisfactory stability and better results than previously reported MXene-based biosensors. This work has potential applicability in the bioanalysis of cTnT and other biomarkers and paves a new path for fabricating high-performance MXenes for biomedical applications and electrochemical engineering.

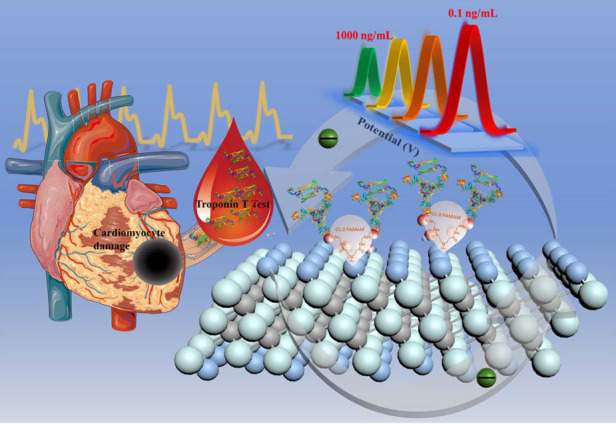

## Introduction

Cardiovascular diseases (CVDs) remain the leading cause of death worldwide, resulting in the loss of an estimated 17 million lives each year^1^. An acute myocardial infarction (AMI), or a heart attack, is one of the most direct life-threatening forms of CVD and accounts for the primary proportion of cardiovascular death because it causes irreversible heart damage and ultimately heart failure^2,3^. Timely and fast diagnosis before the onset of an AMI is essential for attenuating and preventing its progression, which can further improve the survival rate. Current clinical diagnostic tools mainly rely on electrocardiographic activity and dynamic changes in cardiac biomarkers, and the latter is critical in diagnosing AMIs because the sensitivity of the electrocardiogram may be as low as 50% or even lower, especially when patients have unstable angina^4,5^.

Cardiac troponin T (cTnT), a vital regulatory protein with cardiac specificity and sensitivity, has been acknowledged as one of the principal biomarkers for AMIs and can provide a long detection window of 2 to 10 days for myocardial cell injury and death^6,7^. Since individuals at risk of AMIs demonstrate increased cTnT in serum, real-time and rapid quantification of AMI biomarkers can effectively prevent premature death by identifying those at highest risk of AMI and offering timely treatment. Although high-throughput instruments such as Roche Cobas-8000 are in widespread use for cTnT tests of patients admitted to hospitals, these sophisticated instruments are only found in dedicated and centralized laboratories and require a longer turnaround time, rendering them ineligible for active surveillance and point-of-care application. Other commercial portable devices, such as Roche Cobas h232 and Abbott i-STAT, which are capable of monitoring cardiac troponins, have a high-cost burden on patients^8,9^. As a powerful analytical tool, electrochemical immunosensors exhibit high sensitivity, rapid response, good portability, low cost, and easy integration and miniaturization, and there has thus been substantial interest in developing electrochemical immunosensors for cTnT^10–12^. To date, many works on cTnT detection using electrochemical techniques have demonstrated very good performance, yet they are limited by either complicated probes or tedious detection steps^8,13^. Therefore, developing a novel and reliable strategy for constructing a cTnT immunosensor is valuable and important.

One critical aspect of fabricating a high-performance electrochemical biosensing system is to establish a well-defined functional interface, wherein electrode-modified nanomaterials between the transducer and the target recognition layer are beneficial and even indispensable for ensuring and enhancing the sensing ability^14^.

Poly(amidoamine) (PAMAM) dendrimers, orderly three-dimensional (3D) hyperbranched polymers with controllable size and architecture, are commonly known for their hydrophilicity, flexibility, mechanical and chemical stability, and rich surface functionality. In particular, lower-generation PAMAM dendrimers with appropriate molecular weights (less than G4) are regarded as efficient signal amplifiers in biosensing^15,16^. Specifically, the sufficient nitrogen functional groups at their branch end (e.g., amine groups) offer multiple interaction sites for anchoring other metal nanomaterials, especially AuNPs. Additionally, the traditional cores of PAMAM dendrimers can be replaced with high-performance nanomaterials such as graphene to enhance their electrochemical behavior. Replacement results in hybrid heterostructures with modifiable surface functionality, good conductivity, high surface-to-volume ratios, enhanced stability, good biocompatibility, and remarkable molecule-loading capacity^17,18^. Such extraordinary matrices that get the most out of the advantages of each structure and have potentially synergistic effects are ideal electrode materials for sensors to improve sensitivity, stability, and specificity.

MXenes, an important and increasingly popular category of post-graphene two-dimensional (2D) transition metal carbide and nitride materials, have attracted considerable interest in energy storage, catalysis, electromagnetic interference shielding, and sensors^19–21^. Their structure can be defined with the general formula M_*n*+1_X_*n*_T_χ_, where M represents a transition metal such as Ti, Mo, or Nb, X represents carbon and/or nitrogen, T_χ_ denotes the terminated functional surface groups (i.e., F, O and OH), and *n* is an integer typically between 1 and 4^20,22–25^. MXenes have exhibited many extraordinary properties, including great mechanical stability, excellent electric conductivity, a tunable band-gap structure, and excellent thermal properties. More importantly, unlike most other 2D materials, such as graphitic carbon nitride and graphene, MXenes are highly hydrophilic, they have a unique accordion-like structure, (e.g., MXene Ti_3_C_2_T_χ_), and they possess large redox-active surface areas conducive to high-density incorporation of designed functional groups or nanomaterials. These properties make it a superior candidate for electrochemical sensing^26–29^. To date, various MXene-based biosensors have been developed for target detection^30–32^. However, the application of MXenes suffers from stacking and anodic oxidation, although MXenes have desirable charge-carrier mobility and biocompatibility. Such defects result in a reduction in active surface areas for loading biomolecules and lead to the narrowing of an electrochemical window, which eventually has a serious effect on the biosensing performance^33,34^. Notably, to address the inherent limitations of MXenes, several strategies have been employed, including cation intercalation^29^, nanocomposite self-assembly^35^, phase transfer^36^, and interfacial chemical grafting^37^, but applications of these processes to MXenes in electrochemical sensing have rarely been reported. More comprehensive explorations into MXenes are therefore highly expected to broaden and strengthen their applications in electrochemical sensing and biosensor-related fields.

**Fig. 1:**
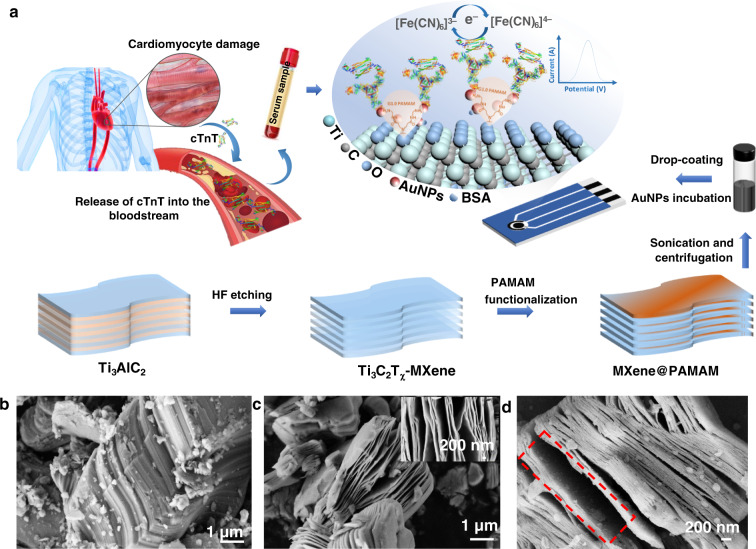
Schematic illustration of the fabrication of the AuNPs/MXene@PAMAM platform and its electrochemical application for cTnT detection. **a** 2D Ti_3_C_2_T_χ_ nanosheets-MXene prepared by acid etching aluminum layers from Ti_3_AlC_2_, were covalently grafted with the first-generation PAMAM dendrimers by in-situ growth method. The MXene@PAMAM supernatant obtained by sonication and centrifugation was introduced onto the surface of disposable SPCE by drop-casting method. Then, the massive amino terminals of PAMAM offered abundant active sites for adsorbing AuNPs to construct 3D heterogeneous nanoarchitecture. Subsequently, thiol-linked antibodies against cTnT were introduced into AuNPs/MXene@PAMAM as the recognition element in the electrochemical immunosensor. The developed cTnT immunosensor were capable of monitoring increased serum cTnT that were valuable indicator of cardiomyocyte damage. SEM images of **b** Ti_3_AlC_2_-MAX, **c** Ti_3_C_2_T_χ_-MXene, and **d** MXene@PAMAM.

In this contribution, to investigate the ultimate potential for using MXenes in biosensing, 2D Ti_3_C_2_T_χ_ nanosheets, typical MXenes, were covalently functionalized with first-generation PAMAM dendrimers by an in-situ growth method using Ti_3_C_2_T_χ_ as the core. The resulting MXene@PAMAM not only inherited structural integrity and superior conductivity of the original MXene but also had a favorable fractal architecture with large specific surface areas, abundant active sites, and improved stability for versatile applications. The introduction of PAMAM to the MXene was expected to simultaneously improve the stability of Ti_3_C_2_T_χ_-MXene and the conductivity of the PAMAM dendrimers, opening a promising new pathway for expanding its application in the electrochemical biosensing field. As a proof of concept, we developed a AuNP/MXene@PAMAM nanobiosensing platform to create a novel and label-free electrochemical immunosensor for the rapid and sensitive analysis of cTnT, which had been proven by our preliminary study^38^. To design such an immunosensor, MXene@PAMAM was introduced onto the surface of disposable screen-printed carbon electrodes (SPCEs) by a routine drop-casting method. Subsequently, AuNPs were self-assembled to construct this 3D heterogeneous nanoarchitecture, which enables a higher immobilization density of antibody, fast target detection, and better electrochemical performance (Fig. 1a). To explore the electrochemical effect of our modifying electrodes, a typical water-soluble redox couple, ferricyanide/ferrocyanide [Fe(CN)_6_]^3−^/[Fe(CN)_6_]^4−^ ([Fe(CN)_6_]^3−/4−^), was adopted as the electron transfer agent in the electrolyte system. The anionic redox probe was used here due to its good electrochemical reversibility, high stability, low toxicity, commercial availability^39–41^, and extensive use in various biosensors, including immunosensors and cell biosensors^42,43^. The combination of this redox system with differential pulse voltammetry (DPV) in our study enabled us to quantify the surface bioconjugation based on the electrochemical surface impedance difference and quickly acquire the generated current signal. This work aimed to provide an opportunity to fabricate high-performance MXene nanocomposites for electrochemical analysis and offer the possibility of overcoming the current limitations of MXene application.

## Experimental section

### Materials and reagents

More details on suppliers for materials and reagents are described in the Electronic Supplementary Information (ESI).

### Apparatus

Details on the suppliers of the instruments are listed in the ESI.

### Synthesis of Ti_3_C_2_T_χ_-MXene

An aqueous acid etching method was adopted to prepare the layered Ti_3_C_2_T_χ_-MXene^20^, and details are presented in the ESI.

### Growth of first-generation PAMAM dendrimers on Ti_3_C_2_T_χ_-MXene

**Fig. 2:**
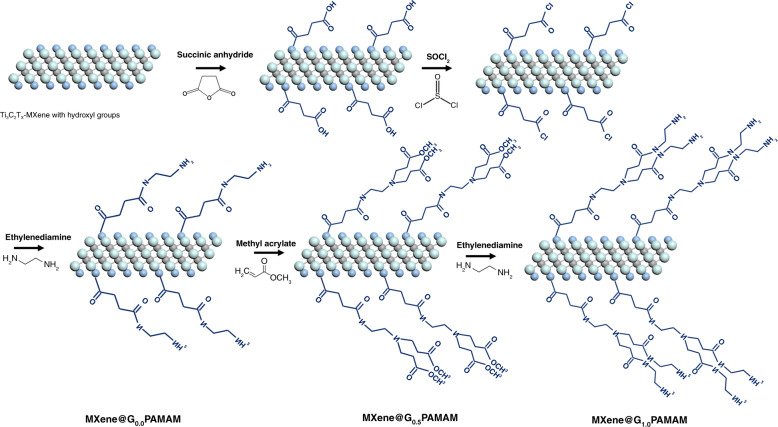
Schematic of the procedure for synthesizing the MXene@PAMAM (abbreviated from MXene@G1.0PAMAM). Ti_3_C_2_T_χ_-MXene featured abundant hydroxyl (-OH) groups that were introduced during HF etching process. These highly active -OH groups easily underwent a ring-opening esterification reaction with succinic anhydride in an anhydrous environment, through which the carboxylic acid functional groups were covalently introduced into the surface of the Ti layer to obtain carboxyl-MXene and for further growth of PAMAM. The as-prepared carboxylates were then reacted with SOCl_2_ to perform acyl chloride reaction, followed by treating with ethylenediamine to obtain the dendrimer initiator MXene@G_0.0_PAMAM. The initiator was then served as substrate and core to accomplish the subsequent reaction of interior branch cells and terminal branch cells. After one cycle grafting reaction, MXene was modified with the first-generation PAMAM dendrimer (MXene@PAMAM).

Figure 2 presents the schematic procedure for preparing first-generation PAMAM dendrimers on Ti_3_C_2_T_χ_-MXene (MXene@PAMAM), and synthetic details are described as ESI.

### Fabrication of the AuNPs/MXene@PAMAM biosensing platform

Prior to this experiment, AuNPs were prepared according to our reported method^44^, and thiol-linked anti-cTnT mAb (SH-mAb) was synthesized as previously reported^41^. Details were given in ESI.

### Electrochemical measurements

Details of the electrochemical measurements are described in ESI.

### Sample preparation

Details on the sample preparation are listed in ESI.

## Results and discussion

### Morphological and structural analysis of MXene@PAMAM

Optional methods for synthesizing MXenes include chemical vapor deposition (CVD)^45^, wet etching^29^, and high-temperature etching^46^. Among them, the typical wet etching strategy has been predominantly employed to deliver high-quality MXenes with rich functional surface terminations relating to their intrinsically hydrophilic nature and electronic properties. Herein, we applied a wet etching Ti_3_AlC_2_-MAX phase to produce Ti_3_C_2_T_χ_-MXene for further PAMAM functionalization, as illustrated in Fig. 1a. After exfoliation, the microstructure changed remarkably from the compact laminated layered structure of the original ternary carbide (Fig. 1b) to the obvious loosely packed accordion-like morphology of delaminating Ti_3_C_2_T_χ_ (Fig. 1c). Typically, Ti_3_AlC_2_-MAX phases featured layered hexagonal crystal lattices, where near-close-packed Ti layers were interleaved with pure Al layers by the Ti-Al bond, and the C atoms filled the octahedral sites between the former, forming Ti-C bonds^20^. The Ti-C bond with a mixed metallic/covalent/ionic character stronger than that of the Ti-Al metallic bond enabled the selective etching of Al by HF, eventually displaying 2D Ti_3_C_2_T_χ_-MXene exfoliated multilayers^47^. Ti-Al bond cleavage and its replacement by Ti-OH/F began at the edges of Ti_3_AlC_2_ through spontaneous intercalation of HF and then spread along the surface with prolonged immersion, hence introducing abundant Ti-OH/F terminations into the entire surface of the MXene, which was favorable to functional regulation^48^. Such rich surface chemistry was superior to that of other 2D nanomaterials, such as pristine graphene, allowing only partial functionalization of the surface defects and edges. The synthesized MXene@PAMAM hybrid is presented in Fig. 1d. Notably, the MXene@PAMAM still retained a representative sheet structure similar to that of the MXene layers, suggesting a lack of significant damage to the structure during the grafting process. Its surface showed obvious roughness, which was notably different from the smooth surface of the MXene (the inset of Fig. 1c); this morphology was probably due to the successful growth of PAMAM units on the MXene.

**Fig. 3:**
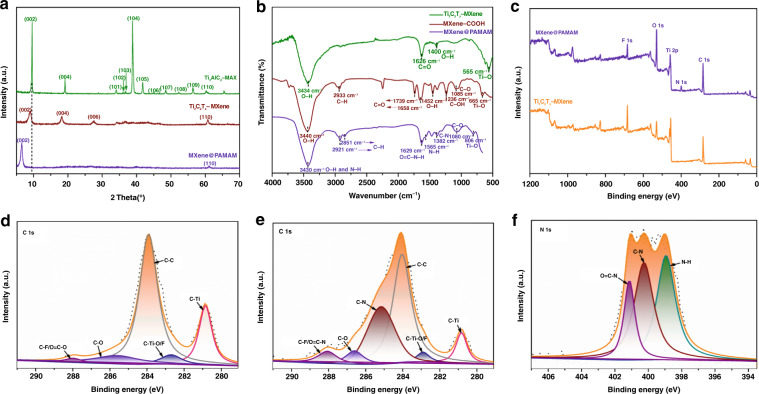
Structural characterization of MXene@PAMAM. **a** XRD patterns of Ti_3_AlC_2_-MAX, Ti_3_C_2_T_χ_-MXene, and MXene@PAMAM. **b** FTIR spectra of Ti_3_C_2_T_χ_-MXene, carboxylic MXene (MXene-COOH), and MXene@PAMAM. **c** Powder XPS survey spectrum of Ti_3_C_2_T_χ_-MXene and MXene@PAMAM. High-resolution XPS spectrum of **d** C 1s spectrum of Ti_3_C_2_T_χ_-MXene, and **e** C 1s and (f) N 1s spectra of MXene@PAMAM.

XRD analysis was used to confirm that the material was prepared. As presented in Fig. 3a, in the XRD pattern from 33° to 45°, peaks of Ti_3_AlC_2_ were identified at 36.01°, 36.77°, 38.82°, and 44.87°, corresponding to the (102), (103), (104), and (106) indices, respectively^49^. The strong peak at (104) indicated the high quality of Ti_3_AlC_2_-MAX, and the diffraction peak at (103) also belonged to well-crystallized Ti_3_AlC_2_^50^. The peak of the (102) facets might be due to the TiC microcrystal, while (106) probably originated from the TiAl impurity, both of which could be caused by the self-propagating, high-temperature synthesis of Ti_3_AlC_2_^50^. After HF treatment, a series of peaks containing aluminum, such as the (104) peak, were obviously eliminated, indicating successful etching. Additionally, the (00 *l*) peaks of Ti_3_C_2_T_χ_-MXene broadened and shifted to angles that were lower than those of the original Ti_3_AlC_2_-MAX phase, revealing the significant loss of crystallinity and an increase in interlayer spacing due to the removal of aluminum from the Ti_3_AlC_2_-MAX phase. Additionally, the 2Ѳ angle of 9.04° in the pattern of the MXene shifted to 6.52° after the interfacial chemical grafting of PAMAM, indicating an increase in the interlayer *d*-spacing from 9.8 to 13.5 Å. This result suggested that the firm anchor of PAMAM on the MXene nanosheets tended to expand the spacing distance between multilayers and prevent these nanosheets from restacking. We also noticed that the characteristic (004) and (006) peaks present in the pattern of the MXene disappeared in the pattern of the MXene@PAMAM hybrid, which was a sign of a distinct reduction in crystallinity owing to the amorphous nature of PAMAM and the close binding within the layers^34^.

FTIR spectroscopy and XPS surveys were carried out to investigate the variation in chemical structures between MXene and MXene@PAMAM. As shown in the FTIR spectrum (Fig. 3b), the strong hydrogen-bonded OH terminals on the surfaces of pristine Ti_3_C_2_T_χ_-MXene were confirmed by the stretching vibration at 3434 cm^−1^ and the bending vibration in the plane at 1400 cm^−1^ ^51^. These highly active −OH groups easily underwent a ring-opening esterification reaction with succinic anhydride in an anhydrous environment, through which the carboxylic acid functional groups were covalently introduced into the surface of the Ti layer to obtain carboxyl-MXene (MXene-COOH) and for further growth of PAMAM. Furthermore, the band at 2933 cm^−1^ observed in MXene-COOH was assigned to the anti-symmetrical stretching vibration of C-H, and more detailed band variation data of the MXene-COOH sample are listed in Table 1. Two carbonyl groups were seen in the spectrum. The peak at 1739 cm^−1^ could be assigned to the ester group introduced by succinic anhydride, and the other peak at 1658 cm^−1^ could be attributed to the formation of the carboxyl group. The peaks at 1452 and 1236 cm^−1^ were the typical absorption bands of carboxyl groups, confirming the presence of carboxyl groups^52^. The peak at 1085 cm^−1^ corresponding to the C-O bond was associated with the ester group. In addition, after ring-opening via the esterification reaction, the stretching vibration of Ti-O at 665 cm^−1^ greatly shifted from its position in the spectrum of the pristine Ti_3_C_2_T_χ_-MXene at 565 cm^−1^, which might have been affected by the conjugated group connected through Ti-O bonds^51^. In the FTIR spectrum of MXene@PAMAM, the peak at ~3400 cm^−1^ shifted slightly compared to its position in the spectrum of the carboxyl-MXene, and we attributed it to the overlap of O-H and N-H stretching vibration absorption. Furthermore, we observed that C-H groups, as common linkages on the carbon backbone, gave rise to two enhanced absorption bands at 2921 and 2851 cm^−1^, implying the extension of the carbon chain and the successful introduction of functional groups. The stretching vibration at 1629 cm^−1^ and in-plane bending vibration at 1565 cm^−1^ were characteristic of the O=C-N-H and N-H groups, respectively^52^. Notably, the carbonyl group in the ester was not observed at ~1700 cm^−1^, possibly because it overlapped with the strong stretching vibration of O=C-N-H. However, the absorption peak of C-O at 1080 cm^−1^ and a similar peak shift of Ti-O (806 cm^−1^) were still observed, demonstrating the presence of the ester group. The above observations clearly supported the successful functionalization of G_1.0_ PAMAM on MXene.

**Table 1:** FTIR peaks at different wavenumbers and the corresponding functional groups.

	Ti_3_C_2_T_χ_-MXene						
Groups	O-H	C=O	O-H	Ti-O	—	—	—
Vibration form	*v*	*v*	*β*	*v*			
Wavenumbers (cm^−1^)	3434	1626	1400	565	—	—	—
	MXene-COOH						
Groups	O-H	C-H	C=O	O-H	C-OH	C-O	Ti-O
Vibration form	*v*	*v*	*v*	*δ*	*v*	*v*	*v*
Wavenumbers (cm^−1^)	3440	2933	1739/1658	1452	1236	1085	665
	MXene@PAMAM						
Groups	O-H/N-H	C-H	O=C-NH	N-H	C-N	C-O	Ti-O
Vibration form	*v*	*v*	*v*	*β*	*v*	*v*	*v*
Wavenumbers (cm^−1^)	3430	2921/2851	1629	1565	1382	1080	806

Powder XPS analysis further corroborated the synthesis of MXene@PAMAM through the identification of the chemical states of the elements on the surface layer. As shown in Fig. 3c, the wide survey spectrum of the pure MXene and MXene@PAMAM hybrid exhibited binding energies of the C 1s, Ti 2p, O 1s, and F 1s orbitals, but an extra N 1s orbital signal near 400 eV could only be found on the latter; this signal stemmed from the functionalization of G_1.0_PAMAM. The C 1s region of the high-resolution MXene spectrum was deconvoluted into five peaks (Fig. 3d). The two dominant peaks at 283.91 and 280.85 eV were assigned to graphitic C-C and internal C-Ti bonds^53^. The other peaks located at 282.70 and 285.60 eV corresponded to the terminal C-Ti (C-Ti-O/F) and C-O bonds at the surface^54,55^. The weak peak at high binding energy of 287.98 eV originated from the C-F and/or O-C=O group^56^. However, after surface treatment (Fig. 3e), the peak intensity of the inner C-Ti layer (280.83 eV) was reduced in line with the coverage of the MXene surface by the grafting dendrimers. A remarkable shift of 0.99 eV to higher binding energy was also observed for the C-O bond; this shift could be attributed to the strong interaction between the hydroxyl group on the MXene and an anhydride molecule^34,57^. Moreover, a single strong peak at 285.16 eV newly appeared, confirming the formation of the C-N bond on the surface, and the surface also showed another characteristic peak at 288.06 eV, possibly contributed by C-F and/or O=C-N moieties^58^. As depicted in Fig. 3f, for the N 1s signal, the deconvoluted nitrogen components at 401.13, 400.26, and 398.93 eV were assigned as O=C-N, C-N, and N-H, respectively^5,59,60^. The presence of C-N and N-H bonds was attributed to amine groups (−CH_2_-NH_2_), while the O=C-N groups could be ascribed to the grafting of ethylenediamine.

To confirm the covalent bonding between MXene and G_1.0_PAMAM, we also performed 1D ^1^H NMR spectroscopy on the MXene and MXene@PAMAM. As shown in Fig. S1, compared to that of MXene, the MXene@PAMAM spectrum had obvious chemical shifts except for the solvent peaks (~3.3 p.p.m. H_2_O and ~2.5 p.p.m. DMSO) and the interlayer Ti-OH terminations of MXene (the blue parts)^61^. From the ^1^H NMR analysis of MXene@PAMAM, a peak at *δ* 7.23 (m, 3H) could be seen in the low field. This peak was the chemical shift of the hydrogen proton on the N atoms connected to carbonyl groups, and its downfield shift was the result of the reduction in electron cloud density, which was caused by the strong electron-withdrawing effect of the N atoms and the π-π conjugation of C=O. In addition, the peaks at *δ* 6.61 (d, *J* = 31.5 Hz, 2H) and *δ* 5.31 (d, *J* = 22.6 Hz, 2H) could be assigned to the hydrogen protons of the terminal amino group, and their chemical shifts in the low field were also affected by the strong electron-withdrawing effect of the N atoms. The peak at *δ* 3.48 (m, 10H) was associated with the methylene hydrogen proton next to the amide group, while the signal at *δ* 2.58 (m, 6H) came from the hydrogen protons of the three methylene groups directly connected to the N atom. Last, peaks at *δ* 2.36 (s, 4H) and *δ* 1.96 (d, *J* = 26.2 Hz, 4H) were observed in the high-field region. Both peaks were the signals corresponding to the methylene hydrogen protons next to the carbonyl group. The ^1^H NMR spectrum of the MXene@PAMAM showed all the expected protons corresponding to the chain of G_1.0_ PAMAM. The above results conclusively confirmed that G_1.0_ PAMAM was covalently attached to the MXene surface by in situ functionalization.

### Characterization of AuNPs/MXene@PAMAM heterostructure

**Fig. 4:**
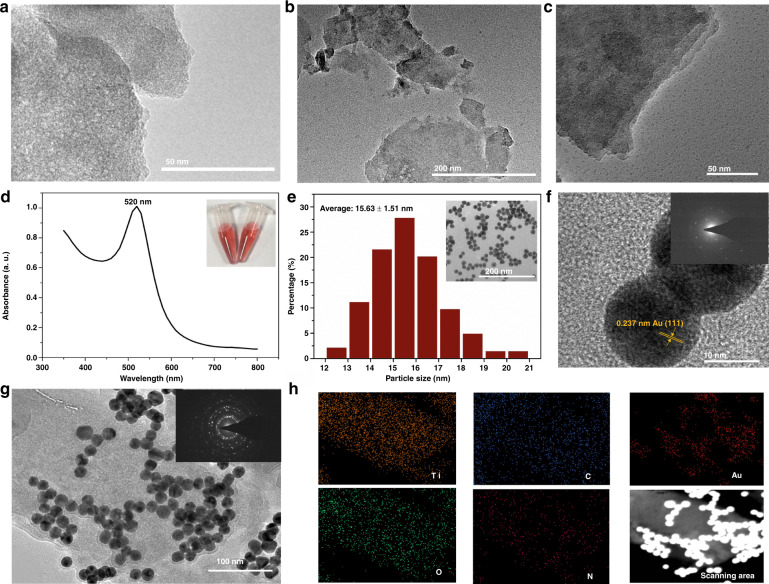
Morphologic characterization of AuNPs/MXene@PAMAM. TEM images of **a** the MXene, and **b**, **c** few-layer MXene@PAMAM flakes. **d** UV–Vis spectrum of AuNPs (inset: photograph of AuNP suspension). **e** Average size and size distribution of AuNPs (inset: TEM images of AuNPs). **f** High-resolution TEM (HRTEM) of AuNPs (inset: SEAD of AuNPs). **g** TEM images of AuNPs/MXene@PAMAM (inset: the corresponding SEAD) and **h** the corresponding TEM-EDS elemental map. The particle size of the AuNPs was measured with the ImageJ software.

The surface morphologies of the prepared nanocomposites were visualized by TEM. As shown in Fig. 4a, the MXene displayed a typical 2D sheet-like structure. After first-generation PAMAM dendrimer functionalization, the morphology of the MXene@PAMAM remained as flat flakes (Fig. 4b, c), but the flakes seemed to be less stacked, which probably stemmed from the modification of the PAMAM dendrimer molecule. In our study, in the in situ growth of these units, the anchoring −OH groups on the MXene probably played a key role in achieving a relatively uniform distribution because the MXene layers served as substrates and cores to accomplish the subsequent reactions of the interior and terminal branch cells. In addition, the presence of the conductive substrate in the hybrid was able to markedly accelerate electron transfer and provide a large specific surface area for seeding dendrimers. The terminal groups of the PAMAM dendrimer molecules endowed the molecules with hydrophilic characteristics, which also gave a reasonable explanation for our observation that the solubility of the MXene@PAMAM in an aqueous solution was better than that of the unmodified MXene. Additionally, the introduction of PAMAM effectively improved the stability and loading capacity of molecules or nanoparticles such as AuNPs given the dendrimers’ intrinsic properties.

Furthermore, to prepare the AuNPs/MXene@PAMAM architecture, we first synthesized AuNPs. The UV–Vis spectrum of the AuNPs (Fig. 4d) featured the characteristic surface plasmon resonance peak at ~520 nm, which conformed to the illustrated color (inset of Fig. 4d). As shown in Fig. 4e, the prepared AuNPs exhibited an approximately spherical shape and had an average diameter of approximately 15 nm. According to the analysis of HRTEM data (Fig. 4f), the lattice fringe spacing of the AuNPs was 0.237 nm, and the AuNPs belonged to the typical Au (111) crystal plane. After facile absorption and self-assembly, the morphological structure of the AuNPs/MXene@PAMAM hybrid was shown in Fig. 4g, where a number of well-shaped AuNPs were successfully attached to the surface of MXene@PAMAM nanosheets. In addition, a relatively uniform distribution of N could be observed in the TEM-EDS elemental map of MXene@PAMAM (Fig. 4h), revealing the successful modification of the first generation of PAMAM dendrimers on the MXene surface. EDS elemental mapping also confirmed the presence of Au. The SAED patterns in Fig. S2 showed that both the MXene and MXene@PAMAM had high hexagonal crystallinity, while the MXene exhibited obvious halo rings and decreased crystallinity after PAMAM modification. This result might be due to the completely amorphous characteristics of the PAMAM dendrimers^62^, and their cover on the thinner MXene flakes easily influenced the MXene crystallinity. The above result was consistent with the XRD results. When the AuNPs were introduced, the AuNPs/MXene@PAMAM hybrid (inset of Fig. 4g) exhibited a clear improvement in crystallinity due to the highly crystalline nature of the AuNPs (inset of Fig. 4f), further verifying the efficient and favorable immobilization of AuNPs on the MXene@PAMAM flakes.

XPS analysis was adopted to characterize the assembly of AuNPs and MXene@PAMAM on screen-printed carbon electrodes (SPCE). As presented in Fig. S3, when compared to that of the bare SPCE, the XPS survey spectrum of the MXene@PAMAM/SPCE showed two additional elements, Ti and N, that were derived from the MXene@PAMAM nanocomposites, and the Cl impurity present in the SPCE might have been introduced during the manufacturing process. In addition, as expected, characteristic peaks corresponding to Au could only be observed after the modification of AuNPs onto the surface of MXene@PAMAM/SPCE^63^, confirming that the AuNPs/MXene@PAMAM/SPCE was successfully prepared.

AuNPs are arguably the most remarkable noble metal nanoparticles owing to their tunable optical properties, distinct electronics characteristics, and unique physicochemical features^64,65^. In particular, several key contributing properties have resulted in the wide application of AuNPs in nanomaterial-enhanced biosensors; these properties include their electrocatalytic ability, easy biofunctionalization, high biocompatibility, and large specific surface areas^65–67^. The active electrocatalytic ability of AuNPs contributes greatly to improving the redox behavior of active species in electrochemical systems and can lead to faster and stronger signal responses. Biofunctionalized AuNPs have shown a strong binding affinity for thiols, disulfides, and amines, which can enable multiple designs of bioconjugation with specific biorecognition elements on the AuNPs (e.g., aptamers or antibodies). The combination of biofunctionalization and biocompatibility further enables highly selective biodetection of the target molecule through affinity binding of specific pairs (e.g., aptamer-protein or antibody-antigen) on AuNPs. Additionally, due to their large specific surface areas, AuNPs are capable of offering available binding sites to increase the density of biorecognition elements, which can critically enhance the sensitivity of a biosensor. Considering the favorable characteristics of AuNPs, in this work, AuNPs were introduced onto the surface of the MXene@PAMAM/SPCE by taking advantage of abundant amine groups on the modified PAMAM. The above characterization results confirmed our successful fabrication of the AuNPs/MXene@PAMAM, and we expected it to exhibit improved electrochemical performance.

### In vitro toxicity of the MXene, MXene@PAMAM, and AuNPs

To investigate the in vitro biocompatibility of the proposed AuNPs/MXene@PAMAM, the cytotoxicity of the MXene, MXene@PAMAM, and AuNPs was evaluated in human foreskin fibroblasts (HFF-1) at various dilution factors of the original working solution (0.5 nmol/L AuNP solution and supernatants of 5 mg/mL MXene and MXene@PAMAM). As shown in Fig. S4, the effects on the survival of the HFF-1 cells were negligible after treatment with the supernatants of the MXene and MXene@PAMAM, even at high concentrations (0-fold dilution of the original supernatant), suggesting the excellent in vitro biocompatibility of the synthesized MXene@PAMAM. The results of the in vitro experiments were similar to those of other reported MXene-based nanomaterials^68,69^, and Lin et al.^68^ suggested that soybean phospholipid-modified Ti_3_C_2_ nanosheets (SP-MXene) showed no noticeable toxicities in vivo, which demonstrated the great application potential of MXene-based nanomaterials both in vivo and in vitro. The AuNPs were found to have no significant cytotoxicity to HFF-1 cells in the range of the tested concentrations by *t* test analysis, although the cell viability appeared to be slightly influenced by the different doses of AuNPs. Our findings regarding the AuNPs were in line with other documented results of AuNP-induced cytotoxicity^70,71^. In summary, cell toxicity in vitro tests of the MXene, MXene@PAMAM, and AuNPs demonstrated the desirable biocompatibility of the three involved nanomaterials and the prepared AuNPs/MXene@PAMAM nanostructure.

### Electrochemical properties of the AuNPs/MXene@PAMAM nanohybrid for biosensing

**Fig. 5:**
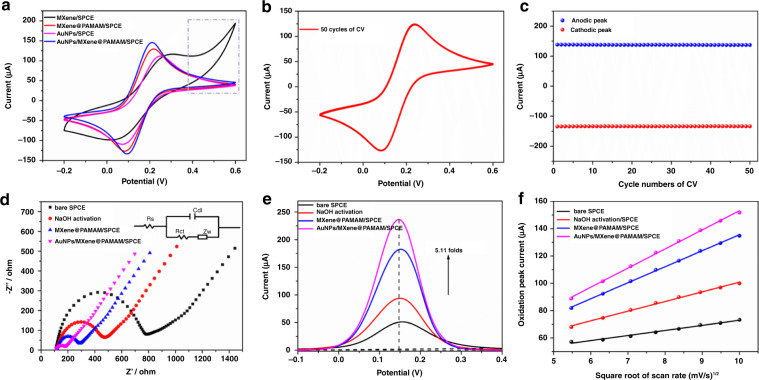
Biosensing properties of AuNPs/MXene@PAMAM nanohybrid. **a** CVs of the MXene, MXene@PAMAM, AuNP, and AuNP/MXene@PAMAM modification on the SPCE. **b** MXene@PAMAM/SPCE with 50 CV cycles and **c** the corresponding redox peaks. **d** Nyquist plots of the bare SPCE, NaOH activation/SPCE, MXene@PAMAM/SPCE, and AuNPs/MXene@PAMAM/SPCE recordings from 0.1 to 100,000 Hz (inset: Randles equivalent circuit of the electrochemical impedance data; Rs: solution impedance, Rct: charge-transfer impedance, *Z*_w_: Warburg impedance, *C*_dl_: double-layer capacitance of the electrode/electrolyte interface). **e** Amplification effect after modification on DPV signal. The modified electrodes were the same as in **d**. **f** Randles–Sevcik plots, the oxidation peak current of the bare SPCE, NaOH activation/SPCE, MXene@PAMAM/SPCE, and AuNPs/MXene@PAMAM/SPCE versus the square roots of the scan rate from 30 to 100 mV/s. All the above experiments were performed in 0.01 M PBS containing 5 mM [Fe(CN)_6_]^3−/4−^ and 0.1 M KCl.

Since 2D Ti_3_C_2_T_χ_-MXene inevitably suffers from irreversible oxidation upon exposure to anodic potentials over 0.2 V, leading to a reduced electrochemical reactive activity and narrow electrochemical window, extending the application of Ti_3_C_2_T_χ_-MXene to electrochemical sensing is a challenging problem^72^. Therefore, in our study, to evaluate the electrochemical stability of the MXene before and after grafting dendrimers under anodic potentials, two types of modified electrodes were prepared by modifying preactivated SPCE with a pristine MXene (denoted as MXene/SPCE) and the synthesized MXene@PAMAM nanocomposites (designated as MXene@PAMAM/SPCE). The MXene was dispersed following the same procedure as was used for the MXene@PAMAM. The two modified electrodes were characterized by cyclic voltammetry (CV) in the presence of a redox couple [Fe(CN)_6_]^3−/4−^ and in potential ranges of −0.2 to 0.6 V as depicted in Fig. 5a. Specifically, the MXene@PAMAM/SPCE exhibited a pair of well-defined oxidation/reduction peaks, indicating the fairly reversible electrochemical behavior of [Fe(CN)_6_]^3−/4−^ on the surface of the electrode. However, atypical redox peaks with significantly decreased current and increased peak-to-peak separation were observed for the MXene/SPCE, reflecting the impediment of the quasi-reversible [Fe(CN)_6_]^3−/4−^ redox process at the liquid-electrode interface. Even worse, its anodic oxidation current density exhibited a sharp increase at higher positive potentials (marked by the gray dotted boxes), and this anomalous phenomenon was caused by the oxidation of the MXene^33,53^. Thus, we inferred that the Ti_3_C_2_T_χ_-MXene had poor electrochemical stability under anodic potentials and less electrochemical activity in response to redox-active reporter molecules, while conversely, the MXene with PAMAM modification was more stable than that without PAMAM modification, and it exhibited improved electrochemical performance. Such a desirable result of MXene@PAMAM/SPCE in the electrochemical system could be ascribed to the fast ion transportation to the electrode interface and the rapid charge transfer to the redox-active center of the MXene in the heterostructure. The robust stability of the synthesized nanohybrid endowed by G_1.0_PAMAM was also beneficial to the electrochemical performance. Furthermore, fifty CV cycles were performed on the MXene@PAMAM/SPCE to investigate its stability. The results in Fig. 5b, c show that the anodic/cathodic currents of the SPCE after MXene@PAMAM modification were almost unaffected by the numerous CV cycles, confirming the good electrochemical stability. The above unique properties made the novel MXene@PAMAM a suitable candidate for electrochemical sensing.

Moreover, the self-adsorption of AuNPs onto the MXene@PAMAM/SPCE resulted in obvious enhancement of the redox current, which stemmed from the excellent conductivity of the AuNPs to accelerate electron transport. To illustrate the loading of MXene@PAMAM on AuNPs, we fabricated a AuNPs/SPCE for comparison with AuNPs/MXene@PAMAM/SPCE. The results in Fig. 5a clearly show that the electrode with MXene@PAMAM and AuNPs exhibited a better current response than the electrode with only AuNPs. This result might be attributed to the large surface area and abundant active sites of the MXene@PAMAM for loading AuNPs, which also indicated the superiority of the AuNPs/MXene@PAMAM system. In addition, to study the loading effect of AuNPs onto the MXene@PAMAM/SPCE, six different SPCEs with AuNPs/MXene@PAMAM modification were prepared using the same preparation method. As shown in Fig. S5, the recorded CVs of the six modified electrodes with an RSD of 1.28% (calculated by oxidation peak current) were almost coincident, indicating the good reproducibility of the AuNPs/MXene@PAMAM/SPCEs.

To reveal the interface behavior of the surface-modified SPCE, EIS Nyquist plots were recorded, as shown in Fig. 5d, wherein the semicircle is associated with the blockage behavior of the electrode/electrolyte interface for charge transfer to the redox couple, and its diameter is equal to the value of the charge transfer resistance (Rct). The plot depicted that the NaOH-activated SPCE had a lower Rct (fitted value 331.8 Ω) than the bare SPCE (fitted value 617.5 Ω), possibly due to the removal of the oxidized layers and other impurities on the surface. More evidently, the SPCE that was preactivated after the successive modification by MXene@PAMAM and AuNPs also possessed a lower Rct (fitted values 157.2 and 64.8 Ω, respectively), implying an improved charge transfer process that resulted from the high electron transmission capability of these two nanocomposites. Similar results were obtained from the corresponding DPV measurement, as shown in Fig. 5e. Moreover, we noticed that the AuNPs/MXene@PAMAM/SPCE achieved remarkably enhanced response signals with a peak current magnification of 5.11 times than that of the bare SPCE, manifesting the favorable electrochemical properties of this functional nanoplatform and the potential availability for biosensing.

The electrochemical kinetics during different modification stages of the SPCE were thoroughly investigated by CV at various scan rates ranging from 30 to 100 mV/s (Fig. S6). As illustrated in Fig. 5f, the anodic oxidation current linearly increased with the square root of the scan rate for all three modified SPCEs, demonstrating that the electrochemical reaction of the [Fe(CN)_6_]^3−/4−^ redox couple on the NaOH-activated and nanocomposite-modified SPCE was a diffusion-controlled process. The obtained kinetics equations were as follows: *I* (μA) = 3.718 (mV/s)^1/2^ + 35.784, *R*^2^ = 0.988 for the bare SPCE; *I* (μA) = 7.064 (mV/s)^1/2^ + 30.182, *R*^2^ = 0.996 for the NaOH-activated SPCE; *I* (μA) = 11.721 (mV/s)^1/2^ + 18.379, *R*^2^ = 0.999 for the MXene@PAMAM/SPCE; and *I* (μA) = 13.984 (mV/s)^1/2^ + 13.255, *R*^*2*^ = 0.998 for the AuNPs/MXene@PAMAM/SPCE. According to the Randles-Sevcik formula *i*_*p*_ = 2.69 × 10^5^ *n*^3/2^*AD*^1/2^*v*^1/2^*c* (where *i*_*p*_ represents the current, *n* is the number of electrons participating in the redox reaction, A is the electrochemical surface area in cm^2^, *D* is the diffusion coefficient of the redox probe in cm^2^/s, *v* is the scan rate in V/s, and *c* is the concentration of the redox probe in mol/cm^3^)^73^, the electroactive surface area of the bare SPCE was calculated to be 0.034 cm^2^, and the three modified SPCEs were calculated to be 0.065, 0.108, and 0.129 cm^2^, respectively, as displayed in Table 2. It was obvious that the real active surface area greatly increased by nearly 300% after modifying the surface of the bare SPCE with AuNPs/MXene@PAMAM, thus effectively improving the electron transfer. This behavior was related to the special 3D heterogeneous nanoarchitecture constructed through AuNP self-adsorption onto the MXene@PAMAM hierarchical hybrid, which provided a high surface area, acted as a 3D conductive skeleton, offered more electrocatalytic sites, and eventually contributed to electron transfer.

**Table 2:** *Rct* of fitted Randles model and values of Randles–Sevcik plots

Modification	Randles model	Randles–Sevcik plots		
	Rct	Slope	*R*^2^	Active surface area
Bare SPCE	617.5 Ω	3.718	0.988	0.034 cm^2^
NaOH activation	331.8 Ω	7.064	0.996	0.065 cm^2^
MXene@PAMAM	157.2 Ω	11.721	0.999	0.108 cm^2^
AuNPs/MXene@PAMAM	64.8 Ω	13.984	0.998	0.129 cm^2^

### Electrochemical characterization of the immunosensor

**Fig. 6:**
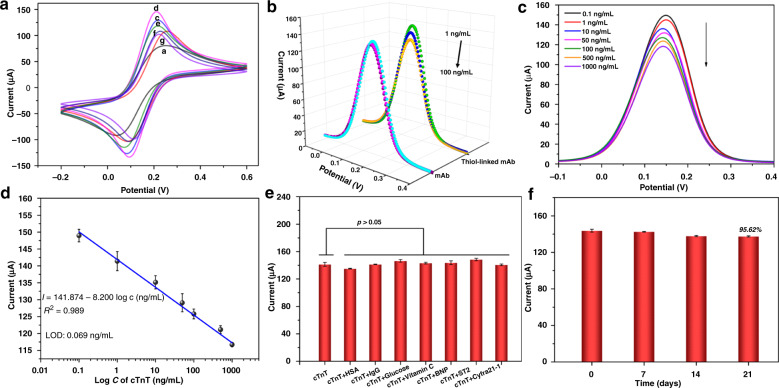
CV characterization and analytical performance of the immunosensor. **a** CVs of a bare SPCE, **b** NaOH-activated SPCE, **c** MXene@PAMAM/SPCE, **d** AuNPs/MXene@PAMAM/SPCE, **e** SH-mAb/AuNPs/MXene@PAMAM/SPCE, **f** BSA/SH-mAb/AuNPs/MXene@PAMAM/SPCE, and **g** cTnT/BSA/SH-mAb/AuNPs/MXene@PAMAM/SPCE. **b** Sensitivity comparison of anti-cTnT mAb- and thiol-linked mAb-based immunosensors using DPV measurement of cTnT antigen (curve from top to bottom: 1, 10, and 100 ng/mL). **c** DPV response of the proposed immunosensor toward different concentrations of cTnT (curve from top to bottom: 0.1, 1, 10, 50, 100, 500, 1000 ng/mL); the peak voltage was ~0.15 V. **d** Calibration curve of the DPV peak currents for various concentrations of cTnT and obtained an LOD of 0.069 ng/mL. **e** Specificity assay of the proposed cTnT immunosensor. **f** Stability study of the proposed cTnT immunosensor. Error bars represented standard deviation (*n* = 3).

CV in ferri-/ferrocyanide solution was performed to characterize the stepwise modification of the developed immunosensor. As presented in Fig. 6a, [Fe(CN)_6_]^3−/4−^ exhibited quasi-reversible one-electron redox behavior after bare SPCE (curve a) was activated by NaOH (curve b) under the optimal activation conditions of −0.6-1.3 V at a scan rate of 100 mV/s for 12 cycles (Fig. S7). Then, given that the MXene@PAMAM was the mainstay for the sensing system, we optimized the coating amount of MXene@PAMAM on the SPCE by tracking the oxidation current response through CV. As presented in Fig. S8, the CV current response continually increased with increasing doses of MXene@PAMAM until it reached a maximum of 12 µL and gradually decreased afterward. A small amount of MXene@PAMAM could not entirely cover the working area (Φ = 3 mm) of an electrode, while excessive MXene@PAMAM was likely to stack together, leading to a reduction in the specific surface area. Therefore, 12 µL of MXene@PAMAM suspension was the optimal amount used to modify the SPCE. Next, we observed that the peak signal was remarkably enhanced with the assembly of the MXene@PAMAM (curve c) and reached a summit after the following step of AuNP adsorption (curve d), which was due to the superior conductivity of the two nanomaterials. Then, the thiol-linked anti-cTnT mAb coating onto the modified SPCE (curve e) resulted in an obviously decreased peak current, probably because an insulating barrier formed by the immobilized proteins hindered electronic communication from [Fe(CN)_6_]^3−/4−^ to the electrode. A similar phenomenon was also observed in the subsequent incubation of BSA and cTnT, and both of the modified SPCEs (curves f and g) yielded declines in the anodic/cathodic peaks. Noticeably, the specific binding between the introduced cTnT and the immobilized anti-cTnT antibody on the surface of the electrode could be accountable for the blocking behavior for the [Fe(CN)_6_]^3−/4−^ redox couple. The above results demonstrated the successful fabrication of the proposed cTnT immunosensor.

### Analytical performance of the immunosensor

The biorecognition mechanism of our proposed immunosensor system was based on the interaction between the antibody and antigen and the effect of a [Fe(CN)]^3−/4−^ redox probe by this interaction, while the transduction mechanism involved differential pulse voltammetry (DPV). As one of the typical voltammetry methods, DPV involves the application of a potential that varies continuously with amplitude pulses on a working electrode, which leads to the oxidation or reduction reactions of electroactive species in the solution (faradaic reactions). These reactions correspond to the adsorption of species, which is dependent on the potential and a capacitive current due to the electronic double layer. Thus, the recorded current is different from the current in the steady state and is related to the process occurring in the working electrode^74^. DPV exhibits higher sensitivity than conventional CV analysis because the current of DPV is immediately measured before pulse application and at the end of the pulse, and the difference between them is recorded. Therefore, in this study, we adopted DPV to measure the response toward various concentrations of cTnT. When the target biomolecules were specifically recognized and captured on the surface of the mAb-SH/AuNPs/MXene@PAMAM/SPCE, an insulating blocking layer that hindered probe diffusion was formed. Such behavior also impeded electron transfer across the electrode/electrolyte interface and eventually led to a decrease in the DPV peak. Therefore, the more protein targets that were captured and immobilized onto the modified SPCE, the more the DPV peak current decreased.

The highly efficient attachment of active recognition molecules to the transducer surface is still the crucial mechanism for ensuring the sensitivity and selectivity of the biosensor. Proper strategies for antibody immobilization are therefore highly expected to deliver an effective biointerface layer suitable for the interaction of antibodies and antigens. It has been proven that uniformly oriented adsorption of antibodies by covalent attachment keeps the antigen-capturing sites distant from the surface-coupling sites and exposes the antigen-binding domains that are more accessible to analytes; such oriented adsorption is more beneficial than the random orientation of physical adsorption^53,75^. In this work, we utilized a thiol-linked anti-cTnT mAb as the molecule recognition element for biosensing. This approach allowed direct immobilization onto the surface of the AuNPs/MXene@PAMAM/SPCE through Au-S bonds instead of via intermediate layers such as self-assembled monolayers, protein A or G. These intermediate layers present an additional obstacle to electron transfer at the interface. Figure 6b shows the DPV measurements, which compared the sensitivity of the anti-cTnT mAb- and thiol-linked mAb-based cTnT immunosensors. The immunosensor prepared by thiol-linked mAb achieved an evidently distinguishable current response toward the three concentrations of cTnT. As a comparison, an almost identical response could be observed with nonfunctionalized mAbs at the three different concentrations. The different results could be due to the efficient immobilization and high alignment of the exposed antibody layer over the electrode surface, which was favorable for recognizing the antigen. Additionally, good orientation may contribute to better electron transfer between the electrode and electrolyte, resulting in relatively high current outputs. In summary, the proposed immunosensor for thiol-linked mAb was more sensitive than the conventional mAb immunosensor.

The analytical performance of the immunosensor was investigated by testing a series of target concentrations. As shown in Fig. 6c, due to the hindrance of the immune complex formed by antigen-antibody specific binding at the interface, the DPV peak current at ~0.15 V gradually dropped with an increased concentration of cTnT antigen. The current drop was plotted against the concentration of cTnT with a linear regression equation of *I* (μA) = 141.874–8.200 lg c (ng/mL), and the determination coefficient (*R*^2^) was 0.989 (Fig. 6d). The limit of detection (LOD) could be calculated as 0.069 ng/mL according to the *M*_b_ + 3 SD (*M*_b_ and SD were the mean value and standard deviation of the blank, respectively)^76,77^.

Furthermore, a specificity assay for the proposed strategy was performed in the presence of 1 ng/mL cTnT mixed with 100 ng/mL of several interfering substances, including HSA, human IgG, glucose, vitamin C, BNP, ST2, and Cyfra21-1. As shown in Fig. 6e, the DPV signal between cTnT with and without interference varied slightly and showed no significant difference (*P* > 0.05), demonstrating that the interference caused a tiny response toward the cTnT immunosensor. The satisfactory specificity of the immunosensor could be ascribed to the intrinsic specificity of the antibody and, more importantly, the antifouling ability of the AuNPs/MXene@PAMAM system. To evaluate the stability of our proposed immunosensor, the prepared BSA/SH-mAb/AuNPs/MXene@PAMAM-modified SPCEs were preserved in a refrigerator at 4 °C for 3 weeks, and their DPV responses toward 1 ng/mL cTnT were tested weekly. As presented in Fig. 6f, after three weeks, 95.62% of the initial current signal response was retained, revealing its good stability. The stability of this sensor was better than that of other reported MXene-based biosensors, and, interestingly, it was even comparable to those of other hybrid 2D nanomaterials; this stability might be derived from the favorable properties of the AuNPs/MXene@PAMAM nanoplatform we constructed, especially the intrinsic stability and good biocompatibility^27,31,53,78,79^.

### Spike analysis and detection of serum samples

To analyze the reliability of the developed cTnT immunoassay for practical application, we conducted intra- and inter-spiked recovery experiments with spiked human serum samples at 6 and 60 ng/mL. In the serum detection test, as shown in Table S1, the intra-assay recoveries were found to be 100.14–102.08%, within the variation coefficient (*C*_V_) of 9.91–10.91%. The intra-assay showed recoveries in a range of 102.90–105.01%, within a *C*_V_ of 17.92–18.46%, indicating that our immunosensor exhibited reliable accuracy and reproducibility in analyzing serum samples. In addition, a further feasibility test of this sensing strategy was performed by adopting the cTnT immunosensor and traditional ELISA method (LOD 6.4 pg/mL) to quantify the cTnT level in real serum samples from six patients. As shown in Table S2, five of the six samples were found to contain cTnT by the ELISA kit, and only four of them were detectable by our sensor because the others were below the LOD. Moreover, Fig. S9 depicts that the results of the fabricated immunosensor correlated well with the standard ELISA kit method (*r* = 0.998), suggesting that the developed immunoassay had robust feasibility and significant potential to detect cTnT in clinical serum samples.

## Conclusions

In summary, we successfully produced MXene@PAMAM by covalently grafting the PAMAM dendrimer to an MXene template. The synergistic effect of the MXene and PAMAM endowed the architecture with good electrical conductivity, large specific surface areas, and, more importantly, admirable electrochemical stability under an anodic potential. The final formation of the AuNPs/MXene@PAMAM nanoarchitecture through self-adsorption was applied to construct the cTnT immunosensor. This functional platform exhibited superior electrochemical performance in detecting the analyte. The proposed sensor for cTnT detection showed desirable stability, high specificity, and good sensitivity, achieving an LOD of 0.069 ng/mL and a wide dynamic range of 0.1–1000 ng/mL. Its reproducibility and feasibility were estimated with serum samples, demonstrating desirable applicability to biomedical analysis. Such desirable results benefited from the favorable structural and functional advantages of the fabricated nanocomposites. This work provides an efficient and reliable solution that broadens the application of MXenes to biosensor-related fields.

## Supplementary information

Revised Supplementary Information-ID-MICRONA0-1917NO-
