# Supplementary material for: Covalently grafting first-generation PAMAM dendrimers onto MXenes with self-adsorbed AuNPs for use as a functional nanoplatform for highly sensitive electrochemical biosensing of cTnT
Source: Microsyst Nanoeng. 2022 Mar 30;8:35. doi: 10.1038/s41378-022-00352-8 (PMC8967855; doi:10.1038/s41378-022-00352-8)
Supplement: Supplementary file 1 — Revised Supplementary Information-ID-MICRONA0-1917NO- [file 41378_2022_352_MOESM1_ESM.doc]

**Supplementary Information**

**Covalently grafting first-generation PAMAM dendrimers onto MXenes with self-adsorbed AuNPs for use as a functional nanoplatform for highly sensitive electrochemical biosensing of cTnT**

Xin Liu^1,2,3,4^, Yong Qiu^1^, Deming Jiang^1^, Fengheng Li^1^, Ying Gan^1,5^, Yuxuan Zhu^1^, Yuxiang Pan^1,6*^, Hao Wan^1,2,3,4*^, Ping Wang^1,2,3,4*^

*^1^Biosensor National Special Laboratory, Key Laboratory for Biomedical Engineering of Education Ministry, Department of Biomedical Engineering, Zhejiang University, Hangzhou 310027, China*

*^2^Cancer Centre, Zhejiang University, Hangzhou, Zhejiang 310058 China*

*^3^State Key Laboratory of Transducer Technology, Chinese Academy of Sciences, Shanghai, 200050, China*

*^4^Binjiang Institute of Zhejiang University, Hangzhou, 310053, China*

*^5^School of Biomedical Engineering, Tianjin Medical University, Tianjin, 300070, China*

*^6^Research Center of Smart Sensing, ZhejiangLab, Hangzhou 310027, China*

*Corresponding author:

Yuxiang Pan, E-mail: panyuxiang@zhejianglab.com.

Hao Wan, E-mail: wh1816@zju.edu.cn.

Ping Wang, E-mail: cnpwang@zju.edu.cn.

***S1*** **EXPERIMENTAL SECTION**

*S1.1* *Materials and reagents*

Titanium aluminum carbide (Ti_3_AlC_2_-MAX phase) was obtained from 11 Technology Co., Ltd. (Jilin, China). Hydrofluoric acid (HF), succinic anhydride, thionyl chloride (SOCl_2_), anhydrous tetrahydrofuran, ethylenediamine, methyl acrylate, and all other chemicals were of analytical grade purchased from Aladdin Biochemical Technology Co., Ltd. (Shanghai, China). N-succinimidyl S-acetylthioacetate (SATA) was obtained from Thermo Fisher Scientific (MA, USA). Chloroauric acid (HAuCl_4_•3H_2_O), trisodium citrate, dimethyl sulfoxide (DMSO), hydroxylamine hydrochloride, amicon ultra-0.5 10 K filters, ethylenediaminetetraacetic acid disodium salt (EDTA•2Na), bovine serum albumin (BSA), and human serum albumin (HSA) were purchased from Sigma-Aldrich (St. Louis, USA). Anti-cardiac troponin T (cTnT) monoclonal antibody (mAb), human IgG, recombinant human cardiac troponin T, type B natriuretic peptide (BNP) protein, growth STimulation expressed gene 2 (ST2) protein, and cytokeratin-19- fragment (Cyfra21-1) were procured from Abcam (Cambridge, UK). Enzyme-linked Immunosorbent Assay (ELISA) Kit for cardiac troponin T Type 2 was provided by Uscn Life Science Inc. (Wuhan, China). Phosphate buffered saline (PBS) used in this experiment was 0.01 M with pH 7.4.

*S1.2 Apparatus*

Cyclic voltammetry (CV), electrochemical impedance spectroscopy (EIS), and differential pulse voltammetry were conducted using CHI 760E electrochemical workstation (Chenhua Instrument Shanghai Co., Ltd. China). TE100 screen-printed carbon electrodes (SPCE, Φ= 3 mm, Zensor Research & Development, China) were used for electrochemical tests consisting of a carbon counter electrode, an Ag/AgCl reference electrode, and a carbon working electrode. Fourier transform infrared (FTIR) spectrum was recorded using Thermo Nicolet 6700 (USA) in the frequency of 4000-400 cm^−1^. The morphological analysis was performed on scanning electron microscopy (SEM, Zeiss Ultra 55, Germany) and transmission electron microscopy (TEM, Tecnai G2 F20 S-TWIN, USA). The selected area electron diffraction (SAED) patterns were also collected by Tecnai G2 F20 S-TWIN. X-ray photoelectron spectroscopy (XPS) was carried out using ESCALAB 250 instrument (Thermo Fisher Scientific) with Al Kα X-rays. X-ray diffraction (XRD) patterns were analyzed with SHIMADZU XRD-700 (Japan) at a scan range of 3-80°. ^1^H NMR spectrum were recorded in DMSO at room temperature on an Agilent DD2-600MHz.

*S1.3 Synthesis of Ti_3_C_2_T_χ_-MXene*

5 g of Ti_3_AlC_2_ powders were immersed in 100 mL of a 40% concentrated HF solution and slightly stirred at 30°C for 24 h to remove the Al layer. Subsequently, the obtained sediments were washed repeatedly by deionized water addition, ultrasonication for 15 min, centrifugation at 3500 rpm for 5 min, and decanting until the pH of the supernatant was closed to 6.0, followed by a further wash with ethanol. To prevent dehydration of the hydroxyls on the surface of Ti_3_C_2_T_χ_, the resulting precipitate was dried under vacuum and low temperature to obtain Ti_3_C_2_T_χ_-MXene with abundant hydroxyl groups.

*S1.4 Growth of first-generation PAMAM dendrimers on Ti_3_C_2_T_χ_-MXene*

The active carboxyl group was introduced into Ti_3_C_2_T_χ_-MXene prior to its functionalization of the first-generation PAMAM dendrimers. First, 1 g of the prepared Ti_3_C_2_T_χ_-MXene and 10 g of succinic anhydride were dispersed in 100 mL of ethanol and held at 25°C for 24 h under a stirring rate of 200 rpm. After the ring opening and esterification reactions between succinic anhydride and -OH group on MXene, the products were rinsed using deionized water and then collected centrifuging at 1500 rpm for 15 min, which was repeated seven times to entirely remove extra succinic anhydride. Finally, the sample underwent vacuum drying at 25°C for 12 h to obtain Ti_3_C_2_T_χ_-MXene functionalized carboxyl groups (MXene-COOH).

1g of the as-prepared MXene-COOH was dissolved in 50 mL of SOCl_2_ with continuous stirring at 70°C for 24 h. The reaction mixture was filtered and washed with anhydrous tetrahydrofuran twice, followed by vacuum drying at 25°C for 24 h. Then, the resultant acyl chloride MXene powder was further dispersed in 100 mL of ethylenediamine under an ultrasonic condition of 5 h at 60°C and stirred for another 24 h. After suction filtration, washing with anhydrous methanol thrice and vacuum drying, the dendrimer initiator MXene@G_0.0_PAMAM was obtained. 0.1 g of dendrimer initiator dispersed in 20 mL of anhydrous methanol was carefully dropped into a solution containing methyl acrylate (20 mL) and anhydrous methanol (50 mL) in 20 min with continuous stirring. The reaction mixture then was held at 40 kHz ultrasonic condition for 7 h at 50°C and stirred for another 24 h. Thereafter, to remove non-grafted molecules and unreacted reactants, the resulting precipitate was rinsed using anhydrous methanol three times and then dried at 25°C overnight to obtain a half-generation dendrimer grafted MXene (MXene@G_0.5_PAMAM). This product was further dispersed in 40 ml of 1:1 methanol/ethylenediamine solution, and reacted for 5 h at 50°C with ultrasonic treatment, followed by stirring for 24 h, filtered and washed thrice with anhydrous methanol to obtain the first-generation dendrimer grafted MXene (MXene@G_1.0_PAMAM abbreviated as MXene@PAMAM). After dried at 25°C for two days under vacuum, the resulting MXene@PAMAM was then dissolved in ultrapure water (5 mg/mL) sonicating for 20 min under N_2_ atmosphere at 25°C and centrifuged for 5 min at 3500 rpm. After centrifugation, the obtained supernatant of MXene@PAMAM nanocomposite was stored at 4°C for further study.

*S1.5 In vitro cytotoxicity assay*

Cytotoxicity assays of the MXene, MXene@PAMAM, and AuNPs were performed using the human foreskin fibroblast (noted as HFF-1, purchased from ATCC). Briefly, 5 mg/mL MXene@PAMAM were firstly prepared by dissolving the nanocomposites in serum-free Dulbecco‘s modified eagle medium (DEME, high glucose, Gibco) with sonication of 20 min under N_2_ atmosphere at 25°C. After centrifuging for 5 min at 3500 rpm, the resultant supernatant was collected and regarded as the original working solution for further cytotoxicity test. The same conditions were used to obtain the supernatant of MXene. Similarly, the stock AuNPs solution was diluted with serum-free DEME to prepare 0.5 nmol/L original working solution. Then, the three kinds of original working solution (0.22 µm membrane filtration sterilization, Millipore) were diluted 0, 1:2, 1:4, 1:8, 1:16 by serum-free DEME to perform subsequent incubation with HFF-1 at 37℃ under 5% CO_2_ in a humidified incubator. After coculture 24 h of HFF-1 with the nanomaterials, a standard Cell Counting Kit-8 (CCK8, DOJINDO) was performed to test the potential in vitro toxicity of MXene, MXene@PAMAM, and AuNPs to cells. A negative control group without nanomaterials and a blank control group without cells were set up at the same time. Cell viability of the treated cells could be calculated as the percentage of average absorbance of each treatment relative to the average absorbance of the negative control group.

*S1.6* *Fabrication of AuNPs/MXene@PAMAM biosensing platform*

To fabricate this immunosensor, the SPCE was firstly activated in 0.1 M deaerated NaOH with cyclic voltammetry (CV) scanning from -0.6 V to 1.3 V at a scan rate of 100 mV/s for twelve cycles (twenty-four sweep segments). And then the activated SPCE was thoroughly rinsed with ultrapure water and dried with nitrogen. After that, 12 µL of MXene@PAMAM suspension was dropped onto the surface of activated SPCE and dried under ambient conditions. Subsequently, MXene@PAMAM modified SPCE (MXene@PAMAM/SPCE) was exposed in the fresh AuNPs solution (12 µL) incubating for 2 h at 4°C. Thereafter, the modified electrode was washed gently with ultrapure water to remove excess material and dried under nitrogen. Next, 10 µL of thiol-linked mAb against cTnT was dropped onto the as-prepared AuNPs/MXene@PAMAM/SPCE and incubated for 90 min at 25°C, followed by blocking unspecific active sites with 0.5% BSA and incubating for 40 min. After rinsing with PBS, the modified SPCE was incubated in 12 µL of cTnT solution with various concentrations at 25°C for 40 min.

*S1.7 Electrochemical measurements*

CV at the potential window of -0.2 to 0.6 V with a scan rate of 100 mV/s was used to characterize the modification process of SPCE. EIS at the open circuit potential of 0.16 V in the frequency of 0.1 to 100, 000 Hz with a 0.005 V amplitude were performed to monitor the electrochemical behavior of SPCE. DPV was recorded for cTnT determination in the potential ranging from -0.2 to 0.6 V at a pulse amplitude of 0.05 V and a pulse width scan of 0.05 s. All the above electrochemical measurements were performed in a deaerated PBS solution containing 5 mM K_3_[Fe(CN)_6_]/K_4_[Fe(CN)_6_] as a redox probe and 0.1 M KCl as supporting electrolyte and carried out in triplicate at room temperature.

*S1.8 Sample preparation*

Clinical serum samples of patients and healthy individuals were provided by Zhejiang University Medical College Affiliated Sir Run Run Shaw Hospital. The study was approved by this institutional medical ethics review board, and all participants signed on written informed consent. The obtained serum samples were simultaneously assayed by a commercial ELISA Kit and our proposed method for a comparative determination of cTnT.

**S2 RESULTS**


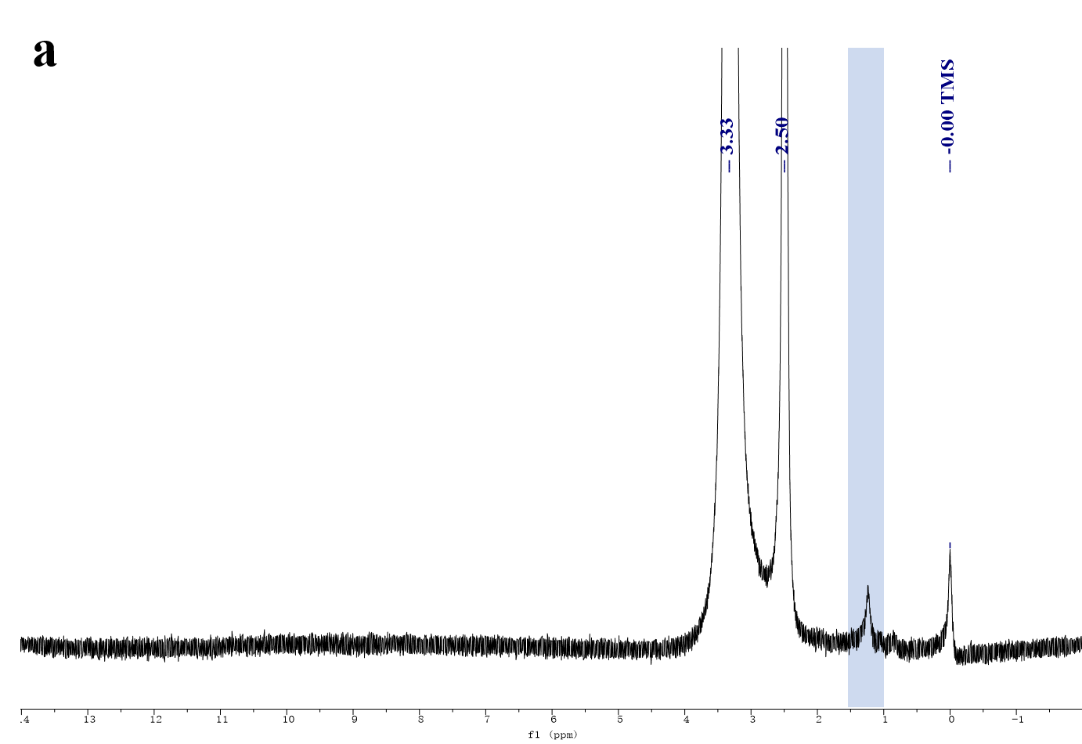


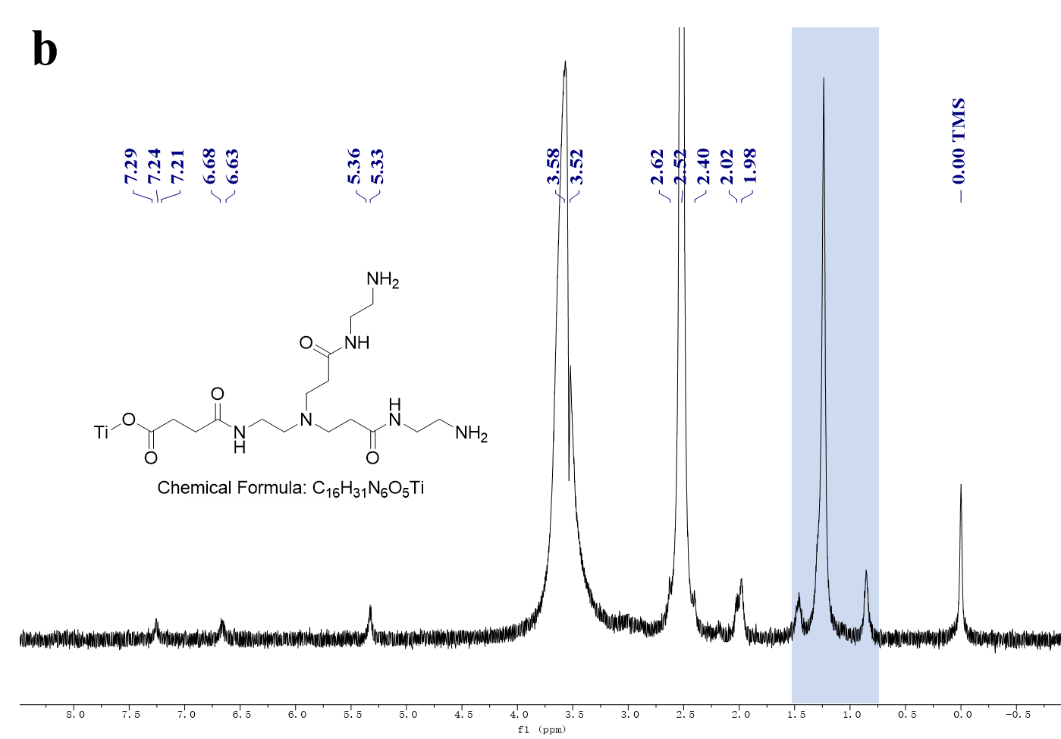


Fig. S1. 1D ^1^H NMR spectrum (600 MHz, DMSO-*d*_6_) of (a) MXene and (b) MXene@PAMAM: δ 7.23 (m, 3H), 6.61 (d, *J* = 31.5 Hz, 2H), 5.31 (d, *J* = 22.6 Hz, 2H), 3.48 (s, 10H), 2.58 (m, 6H), 2.36 (s, 4H), 1.96 (d, *J* = 26.2 Hz, 4H). The bule parts on both of them belonged to interlayer Ti-OH terminations of MXene.

**
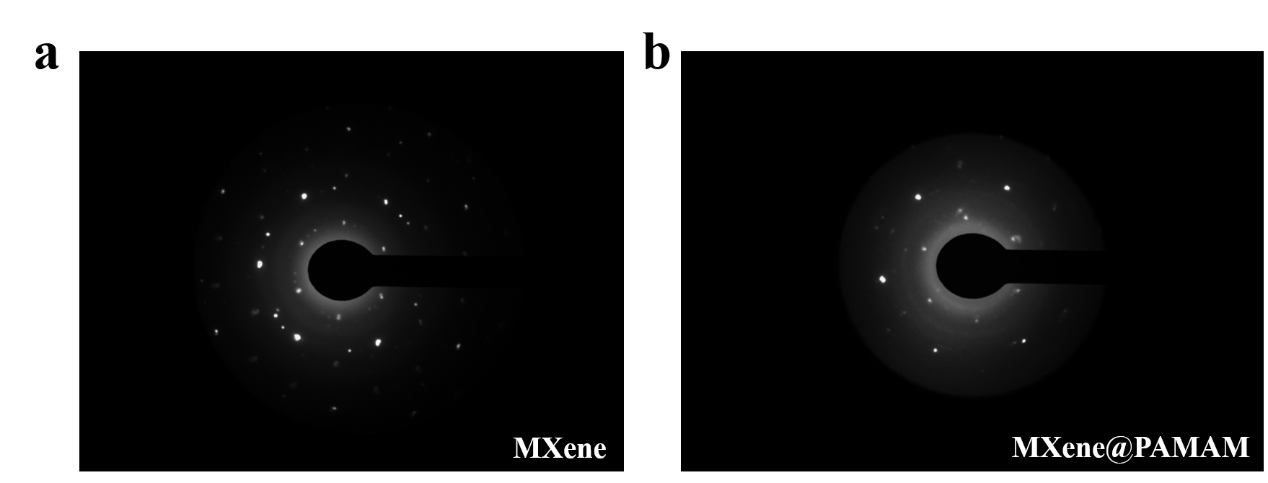
**

Fig. S2. SEAD pattern of (a) MXene and (b) MXene@PAMAM. The SEAD patterns were collected with HT7700 EXALENS.


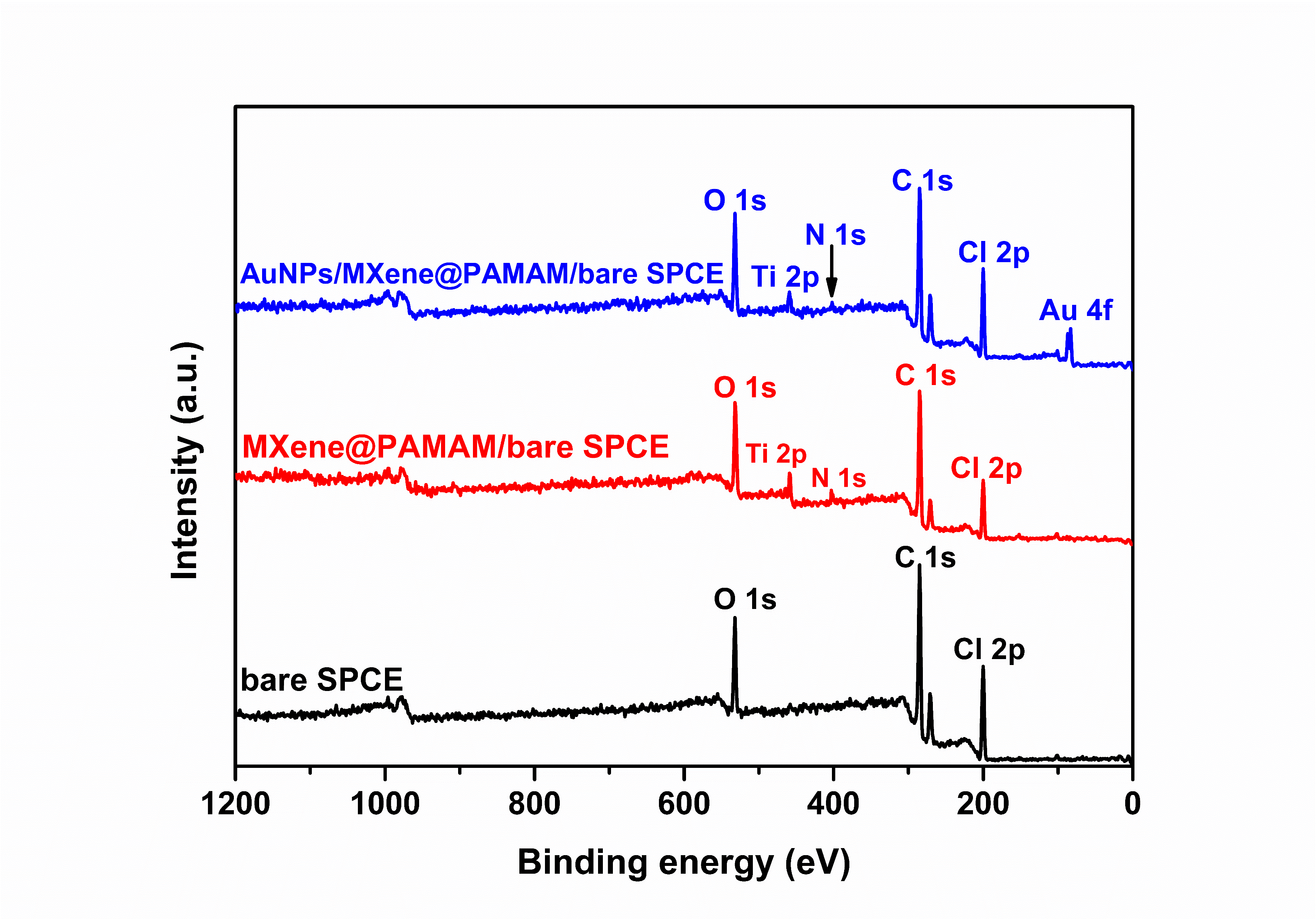


Fig. S3. XPS survey spectrum of bare SPCE, and MXene@PAMAM/SPCE, and AuNPs/MXene@PAMAM/SPCE.

Fig. S4. Cell viability of MXene, MXene and AuNPs in HFF-1 cells at varied dilution factors (0, 2, 4, 8, and 16-fold dilution) for 24 h. Negative control (NC) groups were cultured in a nanomaterial-free medium. Error bars represented the standard deviations (SD) of three parallel samples.


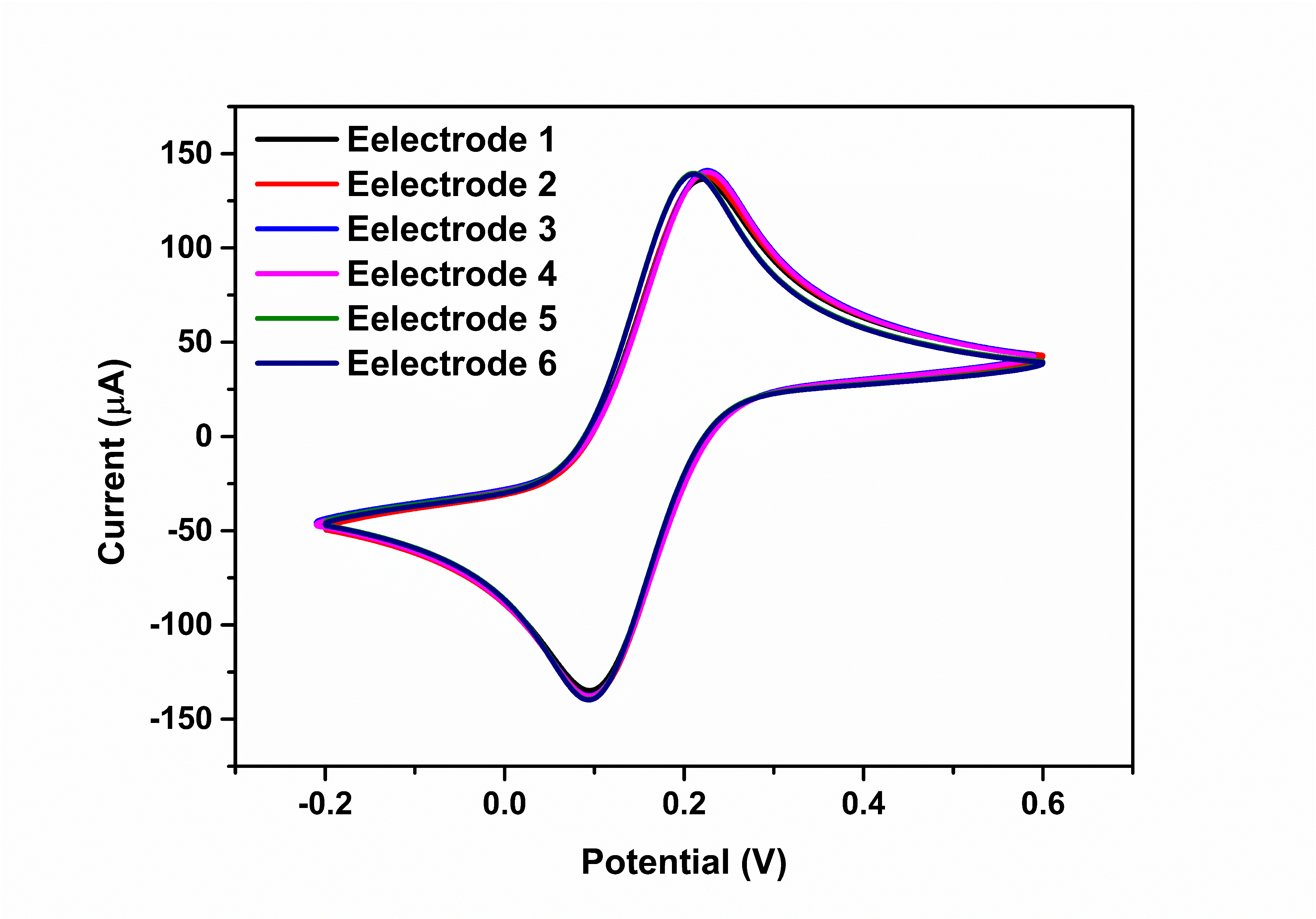


Fig. S5 Cyclic voltammograms of six different SPCEs with AuNPs/MXene@PAMAM modification. RSD calculated by the six recorded oxidation peak currents was 1.28%.


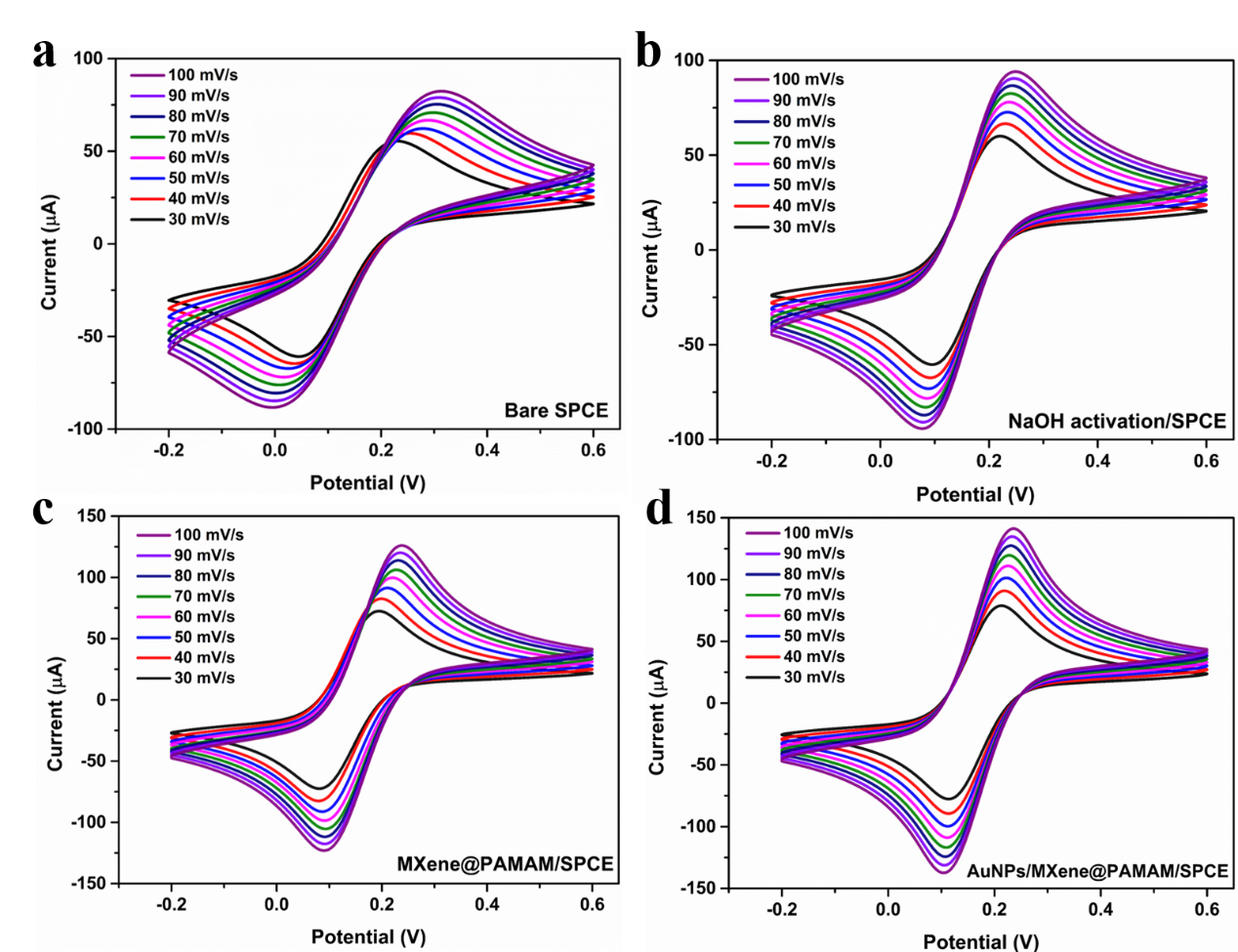


Fig. S6. Cyclic voltammograms of (a) bare SPCE, (b) NaOH activation/SPCE, (c)MXene@PAMAM/SPCE and (d) AuNPs/MXene@PAMAM/SPCE at varying scan rates from 30 to 100 mV/s in 0.01 M PBS containing 5 mM [Fe(CN)_6_]^3-/4-^ and 0.1 M KCl.


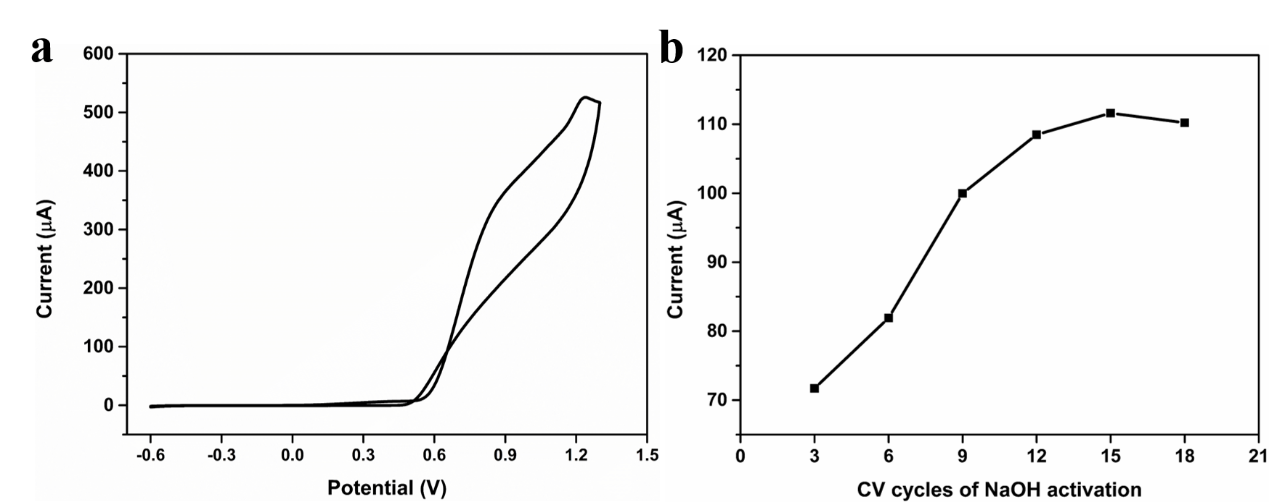


Fig. S7. (a) CV of bare SPCE in 0.1 M NaOH with potential from -0.6 to 1.3 V at a scan rate of 100 mV/s. (b) The optimization of NaOH activation toward bare SPCE. Bare SPCE undergoing NaOH activation under various CV cycles were carried out CV analysis in [Fe(CN)]^3-/4-^ to find the optimal condition. Results showed that the oxidation current signal of CV rapidly ascended with the increased CV cycles of NaOH activation and tended to level off after 12 cycles. So, the optimal parameter to active bare SPCE were 12 CV cycles with a potential range of -0.6 to 1.3 V and a scan rate of 100 mV/s in 0.1 M NaOH solution.


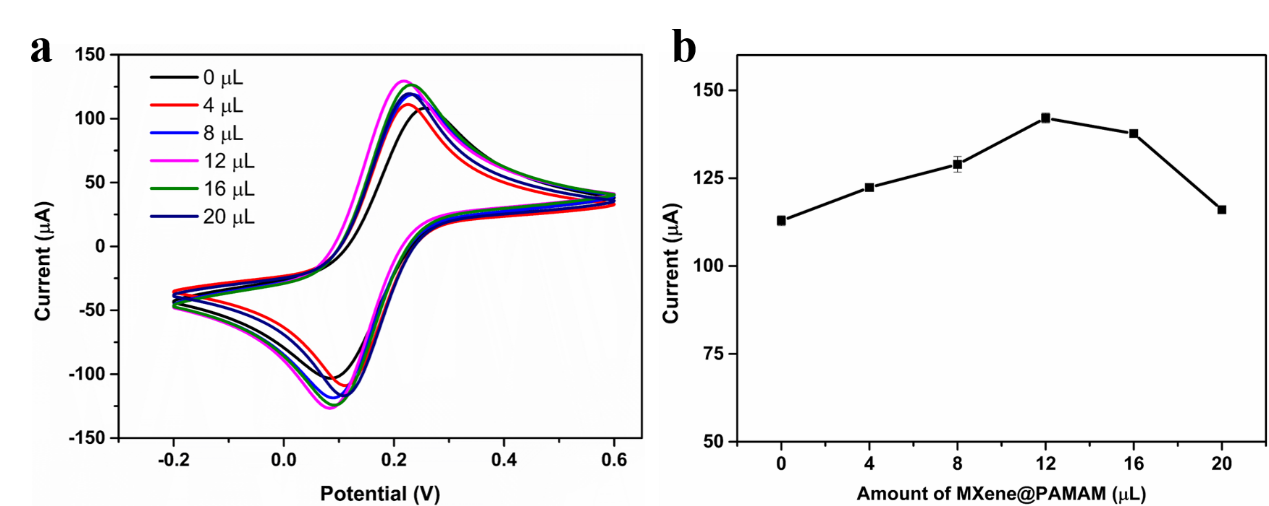


Fig. S8. (a) CV of SPCE modified by different amounts of MXene@PAMAM in probe solution (containing 5.0 mM [Fe(CN)_6_]^3−/4−^ and 0.1 M KCl) at the scan rate of 100 mV/s. (b) The oxidation current signal of CV toward the different amounts of MXene@PAMAM.


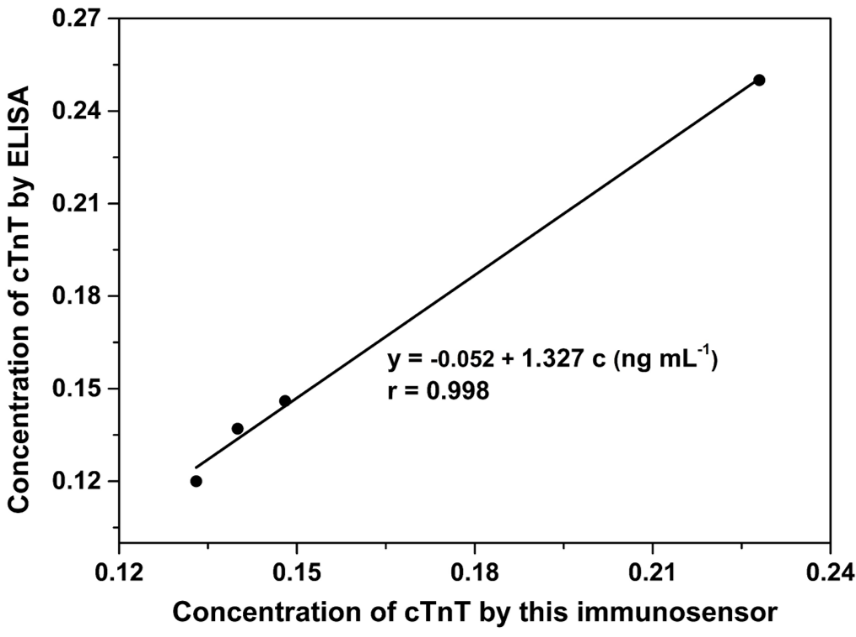


Fig. S9. Correlation between sandwich ELISA and the cTnT immunosensor for cTnT level tests.

Table S1 Recovery analysis of cTnT in healthy human serum sample by the immunosensor

| Sample | Spike level (ng/mL) | Found (ng/mL) | Recovery (%) | CV% |
| --- | --- | --- | --- | --- |
| Healthy human serum ^a^ |  | Intra-assay (n = 3) |  |  |
|  | 6 | 6.008 ± 0.595 | 100.14 | 9.91 |
|  | 60 | 61.250 ± 6.685 | 102.08 | 10.91 |
|  |  | Inter-assay (n = 5) |  |  |
|  | 6 | 6.300 ± 1.178 | 105.01 | 17.92 |
|  | 60 | 61.738 ± 11.396 | 102.90 | 18.46 |

^a^The serum samples were provided by Zhejiang University Medical College Affiliated Sir Run Run Shaw Hospital.

Table S2 Detection result of cTnT content in serum samples by ELISA and the immunosensor

| Serum sample ^a^ | Concentration (ng/mL) | |
| --- | --- | --- |
|  | ELISA (n=3) | Proposed immunosensor (n=3) |
| 1 | 0.0510 ± 0.0007 | - |
| 2 | 0.250 ± 0.010 | 0.228 ± 0.034 |
| 3 | 0.146 ± 0.014 | 0.148 ± 0.024 |
| 4 | 0.120 ± 0.006 | 0.133 ± 0.028 |
| 5 | - | - |
| 6 | 0.137 ± 0.044 | 0.140 ± 0.061 |

^a^ The serum samples were provided by Zhejiang University Medical College Affiliated Sir Run Run Shaw Hospital.
